# Pott’s Puffy Tumor: A Case Report of a Complication of Rhinosinusitis in the Pediatric Age

**DOI:** 10.7759/cureus.84573

**Published:** 2025-05-21

**Authors:** Mariam Noor, Sania Shahid, Nusrath M P

**Affiliations:** 1 Pediatric Emergency Medicine, Al Jalila Children's Specialty Hospital, Dubai, ARE

**Keywords:** frontal sinusitis complication, pediatric emergency medicine, pediatric infection and tumor, pott's puffy tumor, rare presentation

## Abstract

Pott's puffy tumor is a rare and often fatal complication of frontal sinusitis characterized by osteomyelitis and subperiosteal collection of the frontal bone. It is typically managed by surgical drainage combined with antibiotic therapy.

We describe a previously well eight-year-old boy with forehead swelling following rhinosinusitis. A brain CT scan with contrast was diagnostic for Pott's puffy tumor with findings of acute frontal sinusitis with intracranial extension, subperiosteal collection, subgaleal inflammatory changes, and osteomyelitis. The patient was managed medically with intravenous ceftriaxone, vancomycin, and metronidazole, and complete recovery was achieved without surgical intervention.

This case highlights the need to consider Pott’s puffy tumor in the differential diagnosis of atraumatic forehead swelling in children and the importance of treating bacterial sinusitis in this age group.

## Introduction

Pott’s puffy tumor (PPT) is a subperiosteal abscess resulting from frontal skull osteomyelitis. This condition arises infrequently as a complication of frontal sinusitis or forehead trauma and is characterized by tender forehead swelling often accompanied by fever, nasal discharge, headache, or features of raised intracranial pressure [1-3].

Initially, this condition was believed to result from complications arising from direct trauma to the forehead [4]. However, it is now recognized as most commonly occurring due to frontal sinusitis, with young adolescents being the most frequently affected group [1].

The incidence of PPT has significantly declined with the advent of modern broad-spectrum antibiotics for the treatment of rhinosinusitis. Although it can occur in all ages, it is more frequently observed in the pediatric and young adolescent age groups due to relatively underdeveloped frontal sinuses. Early diagnosis by neuroimaging and prompt antibiotic therapy may often prevent the need for surgical intervention and avert potential complications due to intracranial spread, such as subdural empyema, meningoencephalitis, frontal lobe empyema, and cavernous sinus thrombosis [2,3]. This case report details a pediatric patient who has presented to the emergency department with PPT.

## Case presentation

A previously well eight-year-old boy presented to our emergency department with a two-day history of progressive forehead swelling and diffuse headache associated with fever and coryzal symptoms. Two weeks prior to this presentation, he had complained of headache, fever, and nasal congestion and was treated in a different facility as rhinosinusitis with a seven-day course of antibiotics. The parents denied a history of trauma.

**Figure 1:**
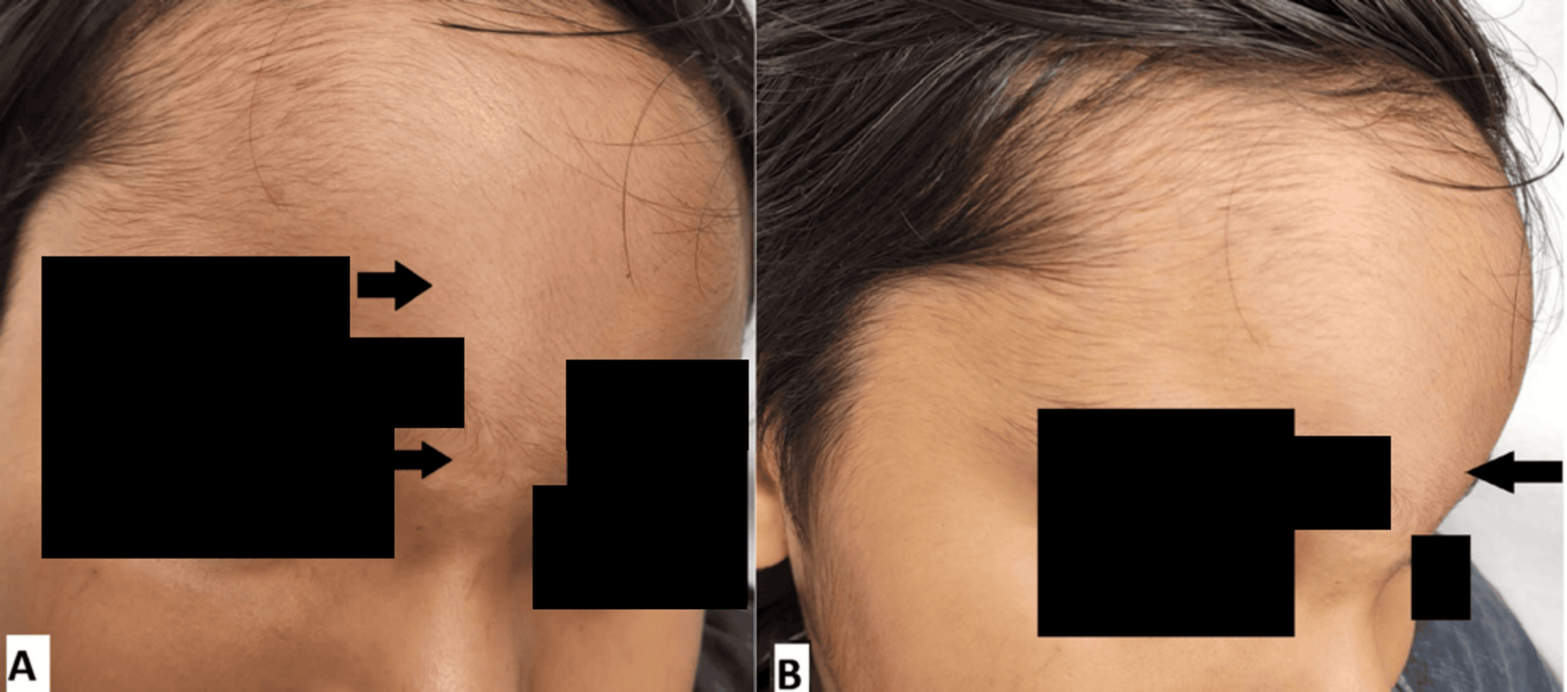
Panels (A, B) show a soft swelling on the forehead extending to the root of the nose. Arrows: swelling measuring 8 x 5 cm

On physical examination, the patient was in pain with a temperature of 38.1 degrees Celsius, heart rate of 131 bpm, blood pressure of 113/65 mmHg, and respiratory rate of 26 bpm. He was alert and oriented. A soft, tender swelling was noted on the forehead measuring 8 x 5 cm and extending to the root of the nose, which was rapidly increasing in size to involve the supraorbital eyelids (Figures 1A, 1B). The remainder of his physical examination was unremarkable.

Blood investigations revealed a white cell count of 12.0 x 10^3^/μL with neutrophils at 79.7%, elevated C-reactive protein (CRP) of 38.4 mg/L, and negative procalcitonin (Table 1). Blood culture yielded no growth.

**Table 1:** Relevant lab results.

Parameter	Result	Reference range
C-reactive protein	38.4 mg/L	0-5 mg/dL
Procalcitonin	0.13 ng/mL	<0.5 ng/mL
WBC count	12.0 x 10^3^	5.0-13 x 10^3^
Neutrophil %	79.7%	40%-70%
Hemoglobin	12.2 g/dL	11.5-15.5 g/dL
Platelet	468 x 10^3^	17-450 x 10^3^

**Figure 2:**
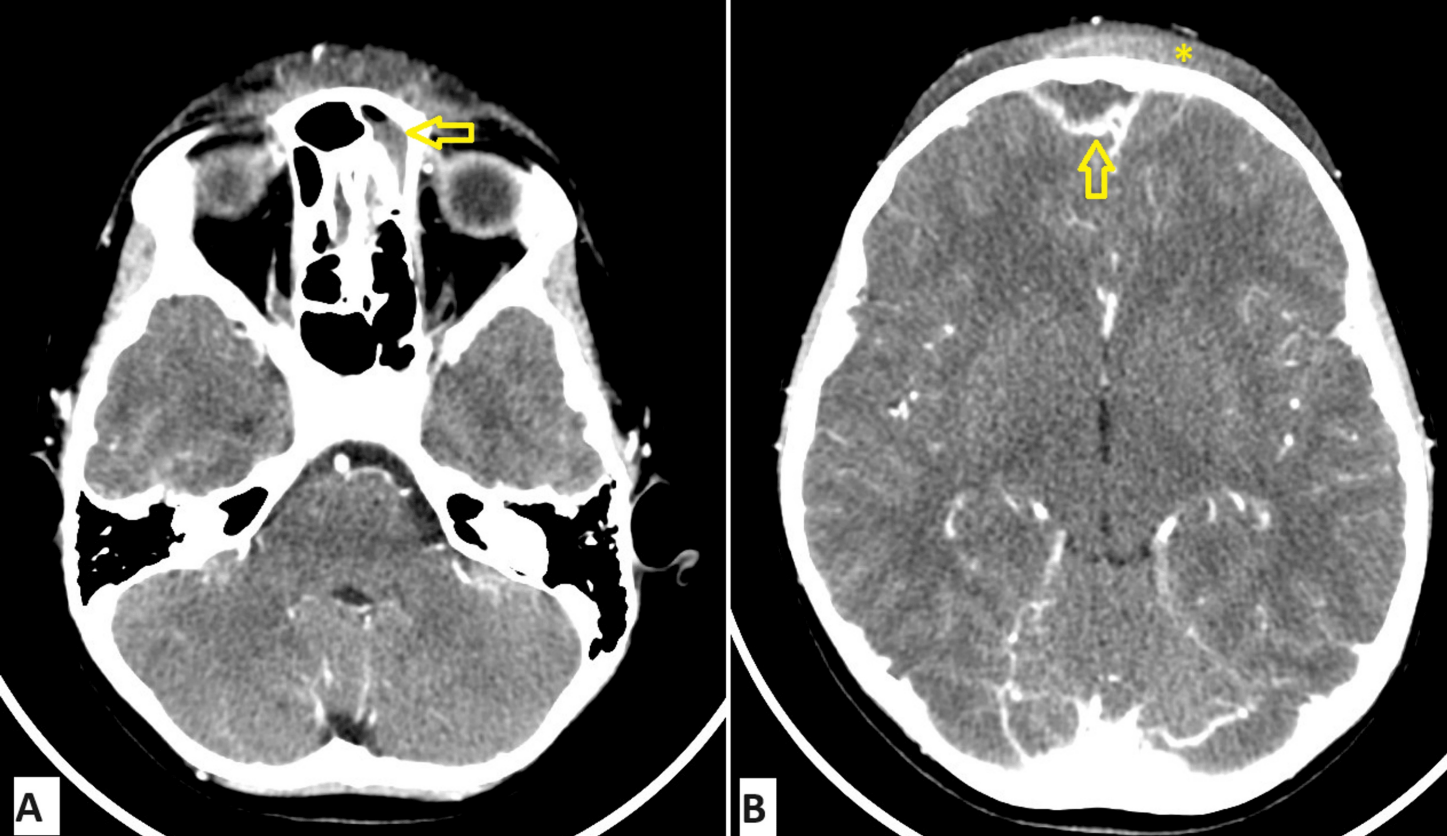
CT brain with contrast done upon presentation. Panel (A) demonstrates soft tissue swelling and frontal sinus mucosal thickening more on the left with a small air-fluid in the left frontal sinus. Panel (B) shows subgaleal and subperiosteal fluid collections and an extra-axial collection at the right frontal convexity with a peripheral rim of enhancement. Horizontal arrow: left frontal sinusitis with air-fluid level; vertical arrow: extra-axial collection measuring 1.2 cm in thickness extending over 3 cm; asterisk: scalp swelling with subgaleal and subperiosteal fluid collections

CT head with contrast demonstrated complicated acute frontal sinusitis with intracranial extension, subperiosteal collection, subgaleal inflammatory changes, and osteomyelitis - findings characteristic of PPT (Figures 2A, 2B). There was no evidence of cerebral venous thrombosis or meningitis.

The patient was admitted and treated by a multidisciplinary team of infectious disease, ENT, neurosurgery, and ophthalmology. Given that the subperiosteal collection was not significantly large, the team opted for a non-surgical management approach. He received intravenous (IV) ceftriaxone, vancomycin, and metronidazole for 18 days. After 48 hours of antibiotic treatment, the child was afebrile with repeat labs showing decreasing CRP of 29 mg/L.

**Figure 3:**
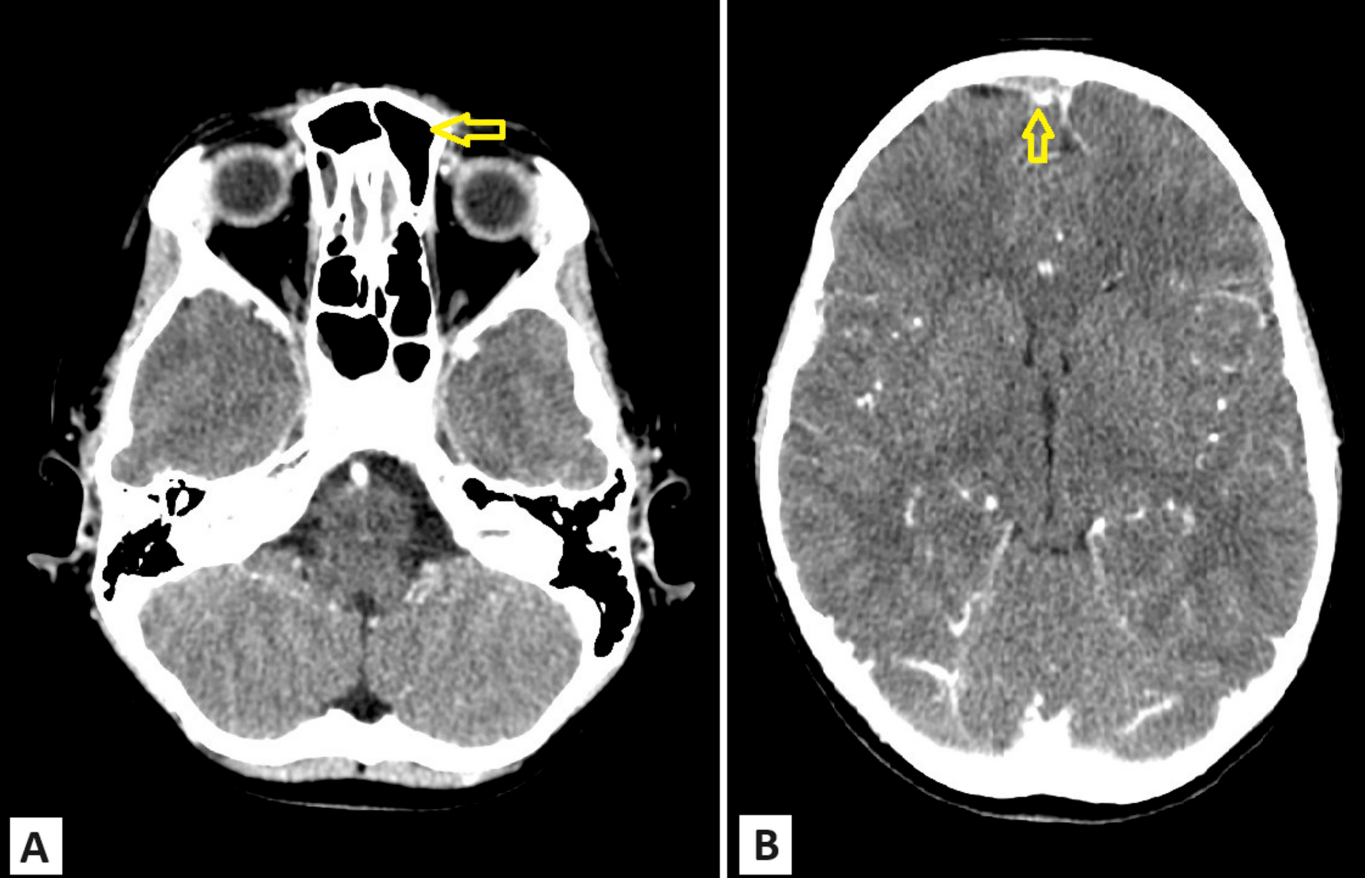
Follow-up brain CT scan with contrast done after two weeks of treatment. Panel (A) shows resolving frontal sinusitis. Panel (B) shows an extra-axial soft tissue lesion in the anterior frontal region with peripheral enhancement, which has significantly reduced in size compared to the previous study. Horizontal arrow: resolving left frontal sinusitis; vertical arrow: reduction in size of extra-axial collection

The frontal swelling and headache subsided gradually during his hospitalization. Serial CT imaging revealed pansinusitis and ill-defined permeative bony erosion of the frontal bone with the resolution of the inflammation and subperiosteal collection (Figures 3A, 3B).

The patient was then discharged home after three weeks of hospitalization on a three-week course of trimethoprim/sulfamethoxazole and linezolid, and complete recovery was observed upon outpatient follow-up.

## Discussion

PPT, described by British surgeon Sir Percival Pott, is a polymicrobial infection. The term tumor refers to one of the four components of inflammation (tumor) and not neoplasia. PPT, also called Pott’s edematous tumor, is a subperiosteal abscess of the frontal bone as a result of osteomyelitis of the frontal sinus walls [5]. Although rare, it may be secondary to head trauma. Episodes of cranial osteomyelitis are considered rare; although a rise in the reported cases has been observed recently, this is most likely because of advances in radiological examinations [6].

Although this condition can affect people of any age or gender, previous studies revealed that PPT in those above the age of 18 years is more common in the 5-17 age range, and they are more common in adolescents, with a median age of 11 years, favoring male patients, who account for 70% of cases [7]. In our case, the child was an eight-year-old boy. In the adolescent age, the flow rate of the diploic veins, which drain the frontal sinus, increases and favors the hematogenous spread of infections [8].

The most frequent cause of PPT that has been documented is either acute or chronic sinusitis, which, if left untreated, may result in bone erosion from the infected material coming into direct contact with intracranial structures [9]. Although less common, craniofacial trauma with a history of fracture is another important cause. Our case had recent sinusitis or upper respiratory infection as the antecedent cause.

The most common clinical manifestations are forehead swelling, fever, nasal congestion, headache, or purulent or non-purulent secretions [2,10,11]. PPT is pathognomonic for a soft erythematous forehead swelling associated with fever [11]. Fever may be absent in some cases, and other symptoms such as orbital swelling, nausea/vomiting, and meningeal symptoms may be present [11].

In our case, the patient presented to the emergency with forehead swelling associated with fever, headache, and coryzal symptoms. He was treated in another facility two weeks back with oral antibiotics for fever, headache, and nasal congestion. CT with contrast demonstrated complicated acute frontal sinusitis with intracranial extension, subperiosteal collection, subgaleal inflammatory changes, and osteomyelitis.

Research indicates that intracranial complications, whether accompanied by bone erosion or not, occur in 60% to 85% of cases [2,12,13]. These complications may remain asymptomatic until advanced stages, especially when affecting silent regions of the central nervous system, such as the frontal lobe [12].

Non-enterococci streptococci (47%), anaerobic bacteria (28%), and staphylococci (22%) are the most common microorganisms [11]. In our case, the culture was sterile.

Surgical management options include endoscopic drainage for early-stage cases, craniotomy for extensive intracranial involvement, and frontal sinusotomy in cases with significant bone erosion or large collections. In more complex cases, a combined approach incorporating both endoscopic and open surgical techniques may be utilized [14,15].

In the majority of reviewed case reports, treatment involved a combination of surgery and antibiotic therapy, with most patients achieving recovery without complications. A combination of surgical intervention and prolonged IV antibiotics has been shown to be the most effective treatment [14]. However, our case had a complete recovery without sequelae after a total of six weeks of antibiotics without surgical intervention. A review of the literature did not reveal any previously documented cases of successful non-surgical management. This case emphasizes the importance of early detection in order to avoid potentially fatal complications.

## Conclusions

A seemingly harmless and common infection, such as acute bacterial sinusitis, can occasionally lead to serious complications. PPT remains prevalent in the pediatric, adolescent, and, less frequently, adult populations, despite its rarity. To prevent fatal complications, it is crucial to recognize and treat benign infections such as bacterial sinusitis. Although surgical intervention is recommended in this condition, early detection can enable a less invasive approach and may even eliminate the need for surgery altogether.
